# An Essay on Curvatures and Distortions of the Spine, and Some Other Morbid Derangements to Which It Is Subject

**Published:** 1823-02

**Authors:** R. W. Bampfield

**Affiliations:** one of the Surgeons to the Royal Metropolitan Infirmary, &c.


Art. II.—*An Essay on Curvatures and Distortions of the Spine, and some other Morbid Derangements to which it is subject.* By R. W. Bampfield, Esq. one of the Surgeons to the Royal Metropolitan Infirmary, &c.

[Continued from No. 285, page 369.]

"What can we reason, but from what we know?"
Pope's *Essay on Man.*

### *Of the Causes and Consequences of Distortions of the Spine.*

Since the days of the celebrated Mr. Pott, there have been laid down by authors, even the most recent, two positions as incontrovertible, relative to spinal disease:—1st, that, wherever there is curvature of the spine, there must be caries; and 2dly, wherever there is caries, there must have existed scrofulous action in the bones of the vertebræ, as the sole cause of disease. These positions are so untenable, that surprise is naturally excited at their having been so long admitted, and acceded to, without a strict investigation of their correctness; for the truth is, curvatures might, with practical benefit, be divided into two species,—curvatures with caries, and curvatures without caries, of which the latter is the most frequent; and the causes of distortions of the spine, being numerous, may, with advantage, be divided into remote and proximate; so that scrofula does not appear to be the *only* cause of carious vertebræ.

Contusions and shocks from falls, sprains of the vertebral joints, scrofula, rachitis, syphilis, rheumatism, careless and habitually bad positions of the body, cerebral affections, and old age, may be enumerated among the remote causes; and, of these, contusions, and shocks from falling have obtained a sort of popular belief of their being common causes, for most parents trace the origin of their children's distortions to nurses or some other persons having allowed them to fall.

The proximate, or immediate, causes should be sought for in the derangement or disease of the structural parts of the bones, intervertebral cartilages, and *perhaps* ligaments, and in lost power of the muscles of the back. Inflammation, ulceration, and caries, terminating in absorption, and destruction of any of the bony parts of the vertebræ; progressive absorption of these parts from pressure; an increased or unequal growth, either of the bodies or of the processes of the vertebræ, producing disproportions between their relative parts, such as are seen in other bones more exposed to view; and progressive absorption, or ulcerative destruction, from any cause, of the intervertebral cartilages, may be considered immediate causes which occasion *permanent* distortion of the spine by mechanical agency. Partial or total paralysis of the muscles of the back, and relaxation of the ligaments of the vertebral column, (*if* the latter do occur to the extent lately insisted upon by Dr. E. Harrison,) will produce *temporary* distortion, that can be at all times removed by mechanical means, as long as the vertebræ preserve their natural and regular dimensions.

It will be of use here to endeavour to trace the connexion between the remote and proximate causes, and to investigate how far the latter are capable of occasioning or leading to distortion; which, perhaps, must be finally inferred to be a pure mechanical effect.

As so many parents attribute the origin of spinal distortions to contusions on the back, from having observed them to be induced soon after such unfortunate accidents, it seems feasible that such occurrences may produce a morbid enlargement, or a morbid destruction, of the bony structures of the vertebræ, by implanting in them a disposition to increased growth and morbid organization, or to inflammation and caries, in the same manner that experience has shown such consequences to follow contusions of the tibia, cranium, and other bones of the body. Contusions on the back may also occasion inflammation and its consequences of the ligaments and invertebral cartilages. Experience supplies convincing proofs that such and similar accidents, as sprains, frequently become an exciting cause, that developes the latent predisposition to scrofula, and calls it into active operation on the injured parts, as in the joints, &c. Independent, however, of any occasional or exciting cause, scrofula may be induced in all or any of the structural parts of the spinal column, and may produce, as dissection has often demonstrated, caries, absorption, and destruction of the bodies and cartilages of the vertebræ, and disease of the ligaments. In some cases of this species, unequivocal marks of scrofula have manifested themselves in the lymphatic glands, or in the joints of the extremities.

Syphilis has been detected as an occasional cause of caries of the bodies of the vertebræ, as well as of disease of the ligaments and intervertebral cartilages, in the same manner that it has displayed its morbid effects on similar structures differently situated. In children, the occurrence of caries from this cause must be extremely rare; but, in adults, no doubt can be entertained of its having been a cause of caries of the vertebræ, and precludes the idea of scrofula being the sole cause.

Sprains of the back will produce inflammation of the ligaments and spinal joints, and weakness of the muscles and ligaments; and will, therefore, give rise to diseased action in those parts, the same as is more familiarly observed in sprains or injuries of other joints; some cases of which we have found on record. They may also lead to distortion, by inducing long continued inclinations of the spine to one side or the other, for the sake of ease.

Rachitis, as far as it affects the osseous structure, may, in a mechanical sense, be defined a constitutional predisposition to unnatural and misshapen formation of bone, and is another immediate cause of spinal distortion, that very obviously produces its *primary* effects by an irregular growth, altering the relative proportions of the bony parts of the vertebræ, which are necessary to preserve their horizontal surfaces on a level, and to keep the spinal column in an upright position. As far as my experience informs me, rickets is the fruitful source of lateral curvatures; and, as yet, I have only witnessed one case of lateral curvature that did not originate in a ricketty habit; and, in almost every instance of spinal deformity from this cause, the bones of the extremities have displayed the unequivocal appearances of ricketty growth and deformed formation.

Mollities ossium has been considered by Mr. Baynton as a remote cause of permanent curvatures of the spine. Should this rare disease invade the vertebræ, it must produce curvature by some part of the bones yielding, on being compressed, so as to alter that proper and relative proportion of the parts of the vertebræ, which is absolutely requisite to the maintenance of the erect attitude.

Rheumatic inflammation occasionally takes its seat in the joints and muscles of the spine, and may produce those changes that lead to distortion, by causing an enlargement of bone and ligament, and a loss of muscular power, the same as it does in similar structures differently situated. It may also occasion distortion, by compelling patients to observe a long continued deflection of the vertebræ from the natural axis.

We have all been taught, in our anatomical lectures, that, as the infirmities of old age steal upon us, the anterior portions of some of the intervertebral cartilages often become absorbed, and occasion the bodies of the vertebræ, at this part of the column, to approximate, and even to anchylose; by which the spinal pyramid is permanently bent forwards, and forms an outward curvature, that may be aptly termed the excurvation of "senility." Long continued position, such as bending forwards or to one side, may produce effects nearly alike in the young.

Great relaxation of the ligaments of joints occasionally occurs in children, more especially of those of the ankles, wrists, and knees; and, in such cases, the ligaments allow of more extensive movements of the bones of the joints, than they admit of in their natural, firm, and almost inelastic state: as, for instance, in the relaxation of the ligaments of the ankle-joint, the foot can be moved in all directions on the lower head of the tibia, to a greater extent than is usual, but more particularly so in the lateral direction; and, if a similar relaxation of the ligaments of the vertebral joints should occur, such partial displacements might at any time be effected as the processes and muscles did not resist and prevent; and, consequently, these luxations must be chiefly posterior. But we have not witnessed any distortions or luxations from this cause; and, indeed, when such should occur, the distortions must be removable at will by mechanical means, just as we can, with the greatest facility, place the foot in its proper position, when, from this cause, it is partially dislocated on either side of the ankle-joint. It may be remarked in a cursory manner in this place, that, in all curvatures, the ligaments situated on the exterior line of the curve must be put on the stretch; but, in this case, the ligaments will be found tense, and not relaxed.

In the generality of cases of spinal distortion, it might be inferred that the energy of the centre of the nervous system is deficient, if such a conclusion may be legitimately drawn from the loss of muscular power that invariably accompanies this disease, ascending from mere deficiency of muscular power to total paralysis and wasting of the muscles; but it is not in the animal powers alone the energy of the brain appears to fail, for the mind becomes subject to despondence, and incapable of employing its faculties with force and activity.

Aneurisms and other tumors occasionally lead to spinal distortions, by the effects of their pressure on the spinal column; but sometimes the pressure of tumors will produce absorption, and even caries, without occasioning curvature.

It has been already observed, that the immediate causes of permanent distortion should be sought for in some morbid alteration or destruction of the structural parts of the vertebral column. This alteration or destruction of structure is effected by "ulcerative absorption," or caries, of the vertebræ; by "progressive absorption" of the vertebræ, without caries or formation of pus; by an irregular growth of bone, combined with progressive absorption; or by either an ulcerative or progressive absorption of the intervertebral cartilages. To effect permanent distortion, this alteration of structure must produce such disproportions in the mechanism of the spine, as not only occasion a deflection from the upright attitude, but are incompatible with its retaining it, and render it impossible to place or restore the spinal column, at once or at will, to its spinal line or natural axis, either by the powers of volition, or by any mechanical power.

Ulcerative absorption (or caries) of the vertebræ and intervertebral cartilages has been certainly produced by scrofula, tumors, syphilis, and very rarely by rheumatic and simple inflammation. Progressive absorption of those parts is produced by tumors in a direct manner, and indirectly by contusions, sprains, rheumatism, bad habits as to posture during the growth of bones, &c. that conduce to this effect by occasioning the body to be for a long time deflected from the spinal line, and thus subjecting particular parts of the vertebral structure to long-continued and undue pressure, which is a very powerful means of exciting absorption. When an increased growth of bone on one part of the vertebræ inclines the spinal column from its straight* line, it occasions increased pressure, and consequent absorption, on the opposite side. Progressive absorption, by a law of nature, frequently takes place in old age, without any extrinsic or evident cause.

The terms temporary and permanent distortion have been employed without their meaning being precisely defined. Permanent distortion must be *perpetual*, where many bodies of the vertebræ are destroyed or taken away, either by ulcerative or progressive absorption; or where, as in old age, progressive absorption of the cartilages occurs, after nature has withdrawn the power of renewal or of depositing new matter. In this essay, however, permanent distortion generally means a distortion not removeable at will or at once, but which admits of the possibility of being ultimately cured. Temporary distortion means a distortion removeable at will, with mechanical assistance. Of this kind are inflections forward from paralysis of the muscles of the back, or relaxation of ligaments. In such cases, the structural parts of the spinal column preserve their relative proportions, and the deflections somewhat resemble the extraordinary curvatures and positions of tumblers and mountebanks.

Caries or ulcerative absorption of the bodies or processes of the vertebræ and cartilages do not necessarily produce curvature, projection, or any distortion of the spine; for, as these latter effects are secondary and mechanical, their production must depend upon *the direction* the ulcerative absorption takes in the destruction of the bony and cartilaginous organization; for, if the ulcerative absorption merely takes away portions of the vertebræ in the perpendicular direction, enough may remain to support the spinal column in its natural attitude, and to preserve its spinal line; but, should the *horizontal* surfaces of the bodies of the vertebræ, with their intervertebral substances, be destroyed or absorbed, as so frequently happens, then the spinal column cannot be placed or supported in the upright attitude;

* Although the word straight is applied to the vertebræ, yet all know the spinal column is not naturally, and strictly speaking, straight.

and if a weight, as of the head and other parts superior to the absorbed portions, be thrown on the spine, it must be deflected from the spinal line; and, should it fall forwards, the spinal processes must be elevated and protrude behind.

To illustrate. Suppose a circular or oval pyramid were erected of many pieces of wood or stone; if a segment of one or more stones, or nearly half a circle, were cut away by the chissel in a perpendicular direction,—or if two segments, of smaller dimensions, be similarly cut away on opposite surfaces, and a central transverse piece be left,—or if parts of the pyramid be cut away all around the circumference, and sufficient of the centre be left,—or if one or more stones be excavated or cut away, so that only three, four, or more, small pillars remain,—still the pyramid would stand, as many antique ruins evince: but if the horizontal superficies of the upper or under side of even a single stone be cut away in an oblique direction, in the shape of a wedge, the pyramid must either incline from the perpendicular attitude to one side, or it must fall altogether. In like manner, anterior, lateral, central, or posterior portions of the bodies of the vertebræ, with their intervertebral cartilages, may be absorbed in the forms and directions we have supposed the pyramid to be cut away, and yet the spinal column shall preserve its erect attitude and spinal line. This fact is clearly established by the evidence dissections have furnished, —by the appearances the preparations in the various anatomical museums present to our view,—and by the plates given of this disease by several authors; and, accordingly, instances of this fact, or *of extensive caries of the bodies of the vertebræ without curvature*, are recorded by Mr. Pott, Mr. Lloyd, and some other authors who have treated this subject systematically; but they do not offer the explanation of the circumstance here given, nor have they particularly mentioned whether the caries destroyed the bone horizontally or perpendicularly: the latter, however, may with safety be inferred, as, in such cases, it is sometimes stated that the caries had more especially destroyed the anterior surfaces of the vertebræ; which destruction, we must suppose, was that of a segment or section,—at all events, not more than a semicircle. It should also be remembered that, besides the mechanical support given by the remaining undestroyed or unabsorbed portions of the bodies of the vertebræ, the muscles of the back, the ligamenta interspinosa, intertransversalia, and capsularia, offer some opposition to any unnatural curvature, and, together with the processes and ligamentum posticum commune vertebrarum,* oppose an almost

* In cases of caries, with or without curvature of the vertebræ, the ligamentum commune anticum is very often destroyed by ulcerative absorption.

insuperable obstacle to the complete luxation of the vertebræ, or the separation of the upper from any inferior part of the spinal column, such as would happen in an architectural pyramid, that has no such collateral supports.

Satisfactory evidence, from the same convincing sources of information already enumerated, will also prove that, whenever curvature of the spine is attended with caries of bone, such caries or ulcerative absorption has frequently been induced on several vertebræ at once, and has always destroyed the horizontal surfaces of their bodies both above and below, not unfrequently in an oblique direction, so that the bodies of the vertebræ (which are carious) gradually diminish in depth from the posterior to the anterior surface, and are reduced into the shape of a wedge,* whilst the intervertebral cartilages are, in general, proportionably absorbed and destroyed, as far as the caries has extended. Hence it is, the spinal column is gradually deprived of the mechanical support that, in the erect attitude, it receives anteriorly; and, when pressure is made from above, by the weight of the head and parts superior to the caries, the spine must incline more and more forward out of its spinal line, in proportion as the horizontal surfaces become more and more destroyed by ulcerative absorption. When the bodies of one or more vertebræ are wholly absorbed, the spinal column is, at those points, completely deprived of all perpendicular mechanical support anteriorly; and its upper part would fall off, or be separated entirely from the lower, (as would an architectural pile thus situated,) were it not prevented by the muscles of the back, the ligaments of the different processes, and the processes themselves, which are seldom involved in the prevailing destruction, all of which concur in preventing the spinal column from being dislocated, even in the most extensive state of ruinous disease.

When progressive absorption of the bodies of the vertebræ and intervertebral cartilages takes place, without caries or the formation of pus, however it may be induced, the same mechanical results and structural deviations must follow, as if they had been destroyed by caries or ulcerative absorption; which will be exemplified in Miss Jane Archer's case, related in this paper. The curvatures arising from progressive absorption generally occur in children during the growth of their bones, when the osseous structure is not very solid: the formation of the curvatures is gradual, and their extent must necessarily be proportioned to the quantity of the horizontal surfaces, or the number of the vertebræ, absorbed. This should teach parents to observe the attitudes of their children, and to endeavour to

* See plates in Mr. FORD's Treatise on Diseases of the Hip-joint, &c. &c. and some admeasurements given of such vertebræ further on.

check or counteract any bad habit, as to the postures of the body, that tends to throw unequal pressure on any particular part of the spinal column.

From some wise arrangement of our preservative powers, which cannot be comprehended or explained, although we can perceive and admire them, it is so ordained by the all-wise Creator that parts, which enclose and defend organs or parts highly essential to life, possess either a higher vitality, or are endowed with a stronger capacity or power of resisting diseased actions and their effects, than those of similar structure which discharge no such office. We could not but premise this observation to the fact, however it may be explained by others, that the posterior surfaces of the bodies of the vertebræ, which form the anterior part of the spinal canal, are generally the last to yield to, and be destroyed, by ulcerative or progressive absorption; whilst we have not yet witnessed an instance of the posterior part of the spinal conduit formed by the processes being wholly absorbed, or even of its continuity being destroyed, although we have seen the structure much altered, and nature (as it were) reduced to the necessity of uniting several spinous processes by anchylosis, to maintain a continuity of the canal, and ensure a competent, or even moderate, defence to the spinal marrow. When the bodies of the vertebræ are wholly destroyed by progressive absorption, we have seen the ligamentum commune anticum much thickened and partly ossified, and anteriorly covered with fat, in order to protect the medulla spinalis. Thus, in the destruction of the vertebræ from progressive absorption, in cases of curvature without caries, it is seen that the ligamentum anticum commune vertebrarum is preserved; whilst, in the destruction of the vertebra from ulcerative absorption or caries, it has been already remarked that this ligament is destroyed,—a circumstance evincing some difference in the nature of the two affections. In such cases the calibre of the spinal canal, and the circumference of the medulla spinalis, are equally diminished in size in that part of its course where the bodies of the vertebræ have been absorbed, as in Miss J. Archer's case.

The part of the subject next to be discussed may be premised by an illustrative observation, that if a wedge, or wedges, be driven between two or more divisions of an architectural column, they must, after first raising and gradually inclining it from the perpendicular to one side, finally overthrow it. As long as the constituent parts of the vertebral column accurately maintain their relative proportions, agreeably to their natural formation, no permanent curvature or angular projection can take place. If, in this state, a wedge were applied between any two vertebræ on any side, the spinal column would be forced out of its spinal line, and incline to the opposite direction. Thus, if it were applied posteriorly, it would cause it to deviate from its spinal line, and bend forwards, and *vice versa*; and if the wedge were driven in between two vertebræ on the right side, it would occasion the column to incline over to the left side, and *vice versa*; and, were it not for the retaining powers of the muscles, ligaments, &c. the spinal column would be dislocated or overthrown altogether. In this point of view, as well as in those we have already taken of the effects of ulcerative or progressive absorption of the horizontal surfaces of the vertebræ, permanent curvatures or angular projections of the spine are merely mechanical consequences; and if we can demonstrate that a power equal to, and acting on the principle of a wedge, be applied in such cases as a cause, we shall prove the position just laid down. Here we must recur to the old exploded doctrine of unequal growth of bone, which has been assumed by Glisson and others as an efficient cause of distortion; a doctrine exploded, too, against the evidence of the most perfect of our senses: for, in the anatomical museums, specimens of deformity arising from the unequal and irregular growth of all the bones, and of course of the vertebræ, may be seen; in some of which the inequality is produced by diseased actions, but in others simply by an exuberance of the "formative principle," or by an error of nature in exciting a pruriency of growth. It can only be intended, however, to offer illustrations of the effects of this error of nature in the vertebral column; and this we are enabled to do in a manner that appears satisfactory, by resorting to the appearances which dissection discloses in H. Pittam's case, in the first part of this essay, in the London Medical and Physical Journal for November, and by examining the preparations of the various distortions of the spine that are placed in the Hunterian, Brookes', and other splendid collections of morbid anatomy. In viewing the preparations, and in the dissection, of spinal deformities from irregular growth of bone, the disproportions of the different bodies and intervertebral cartilages, that is, between their outer and inner lines of curvature, are most striking; the thickness of the vertebra being sometimes, on the outer line of curvature, double, or even treble, of that of the inner line. It therefore remains to be explained by what process this immediate cause produces its consequences.

When an increased growth of bone takes place on one side of the spinal column, which is flexible, it must necessarily elevate the parts above it on the same side, and make them recline over to the opposite side, which is thinner, and that must now sustain the greater share of weight; one consequence of which must be, that a greater degree of pressure will be made by all superincumbent weight on the thinner side, to which it is reclined, whilst it will be diminished on the side which the increased growth has elevated: and, indeed, whilst the superincumbent weight will press the surfaces of the joints more closely together on one side, it will tend to open the joints or separate their surfaces on the other, and put the ligaments on the stretch, and (as it were) create a space for interstitial deposition. Now pressure is the strongest power we possess of producing local absorption, and, as increased growth of bone is a cause of a permanent nature, the pressure too becomes permanent, (except in particular positions of the body,) and occasions absorption of that part of the bone and of the intervertebral cartilage unduly pressed upon. Thus, increased growth on one side, and progressive absorption occasioned by pressure on the opposite side, both conspire to the same end, and both operate in destroying the natural proportions of the vertebræ, and of deflecting the erect spine from its spinal line to the form of a curve; and, in the degree that increased growth takes place on one side, and increased pressure and absorption on the other, the greater or less must be the curvature. Besides, as these causes extend their effects, the number of the vertebræ involved in the curve will be increased, until many are bent into an arc, some of which have had their dimensions increased on one side, and all of them have been diminished on the other,—which is a very important fact to be borne in mind for our guidance in the treatment. During this deforming process, we may reasonably believe the muscles and ligaments offer some resistance to its progress, but effects show their resistance is overcome; and, indeed, it might be observed, on the authority of Mr. Pott, that the ligaments do not always resist the process of deformity, as he found them "somewhat relaxed, thickened, and altered."* Mr. Pott speaks generally, as if all the ligaments were relaxed; and it is to be lamented he did not state the particular ones so affected, &c. as our dissections have not as yet disclosed the state of ligaments precisely as he describes them.

The explanation above offered will be deemed satisfactory, as far as it relates to the manner in which lateral curvature is produced by an irregular growth of bone, even on the most superficial consideration, and perhaps as far as incurvation arises from this cause; but it may not be considered so striking an elucidation of the mode by which the excurvations and angular projections of the spine are induced. Let us then examine how permanent curvatures outwards, or excurvations, are produced. By H. Pittam's dissection, and in some anatomical

* Pott on Palsy of the Lower Limbs, *loc. cit.*

preparations of curved spine, it will be seen that a morbid enlargement, or increased growth, of the transverse and spinous processes, or of " the bony bridge" of some vertebræ, occasionally occur; the mechanical effects of which are varied and progressive. First, the enlarged bony bridge presses on the posterior part of the vertebra immediately below it, and sometimes occasions more or less absorption of it; secondly, it must elevate, like a wedge, the spinous processes above, and occasion them to protrude from the spinal line, and form the angular projection;* thirdly, if the growth increase, then, in proportion as the posterior portions of the vertebral mechanism become further enlarged, must the spinal column be eventually inclined forward more and more, and the weight of the body above, which, in the natural and perfect attitude, is equally divided or borne by the horizontal surfaces of all the vertebræ, will be thrown in an unequal degree upon the anterior surfaces of some of them, and occasion unnatural pressure. Hence, two circumstances will conduce to the formation of excurvations: one is the continually increasing enlargement of the posterior portions of some vertebræ; the other is the absorption of the anterior portions from mechanical pressure. Besides this effect on the anterior portions of the vertebræ, there is also a particular effect produced by the general derangement on that vertebra immediately inferior to those involved in the curvature; for, by the deflection forwards, the superincumbent weight of the head and other parts above the curvature is not equally borne by all the vertebræ, as when they preserved their proper axis or spinal line, but the weight must be thrown on the posterior portion of that inferior vertebra which still preserves the spinal line; and, if it be not so very firmly fixed as to be enabled to retain its situation until it is partly relieved from pressure by the absorption of the vertebræ above, it must be inclined backwards or outwards, as will others in succession similarly situated, and add to the extent of the curve: and thus we see the vertebræ, both above and below the first vertebra, observed to be protruding, gradually formed into a curve; but if the vertebra immediately below those involved in the curvature be firmly fixed, and equal to support the superincumbent weight without being displaced, it becomes the extreme of the chord line of the curvature. The latter is frequently the case with the fourth and fifth lumbar vertebræ, the former of the dorsal, they being the most subject to excurvations. In the specimens of excurvation in Mr. Brookes's museum, attended with destruction of some of the bodies of the vertebræ, the vertebræ and interver-

* One case has occurred to us where " the bony bridge" of two vertebræ were enlarged and projected, without elevating the spinous processes above, or occasioning them to project.

tebral substances below the curvature are thinner than natural on the posterior surfaces of their bodies, where the superincumbent weight has been wholly thrown, and thicker on the anterior, which have been relieved from superincumbent weight by the absorption of the bodies of the vertebræ above.

In those cases it is found that some interspinous ligaments are more or less *elongated*, (a term we prefer to relaxed, as it conveys a more precise meaning of their condition, for they are stretched from being on the outer line of the curve, and are rather tense than relaxed,) and some spinous processes separated at unusual distances from each other, in proportion to the extent of the curvature; for when an elastic body of some thickness is bent from the straight line into the form of an arc, the outer line of the arc or circle, and portions contiguous to it, must be put on the stretch, whilst the inner line of the arc or circle, and portions adjoining, must be compressed; but, if a body thus bent should consist (as in the case of the spine it does,) of different substances, one elastic and compressible, and the other inelastic and incompressible, the first must be altogether stretched on the outer line of the curve, and the most compressed, or at least yield most to compression, on the inner.

By this compression, however, being applied to the inner line of curvature,—that is (in excurvations) to the anterior portions of the vertebral column,—it acts primarily on the elastic and compressible intervertebral cartilages; and hence it is that they are, in general, first carried away by the absorbents, and the bony parts secondly: at least, such is the legitimate deduction from dissections; for, wherever the horizontal surfaces of the bony parts, or the bodies of the vertebræ, have been partly destroyed by ulcerative or progressive absorption, the intervertebral substances have been nearly or wholly so. It has, indeed, sometimes happened that the ulcerative absorption has not removed much of the cartilages, when it has destroyed much of the bodies of the vertebræ in the perpendicular direction; whilst, on the contrary, it has occurred that progressive absorption has removed the intervertebral cartilages, and left the bones entire; of which circumstance a plate is given in Mr. Copeland's Observations on Diseased Spine, and a specimen may be seen in Mr. Brookes's valuable museum,* and of which familiar instances are met with in the curvatures of senility.

* To Mr. Brookes, the learned professor and unwearied cultivator of the sciences of anatomy, surgery, and natural history, the author begs to express his most cordial thanks for the true and most liberal spirit of science with which he promoted his investigations, by placing all the specimens of morbid anatomy of the spine in his museum at his disposal, and by aiding his examinations with the most instructive information and hospitable conduct.

In cases of curvature, progressive absorption of parts must be in the ratio of the compressing power to which they are subjected; and thus the compressed parts will be absorbed and diminished in thickness in the ratio of the compressing power they sustain, and of their degree of liability to absorption. In some constitutions this absorption will go on without inflammation or ulceration being excited by the pressure: at other times, in less fortunate constitutions, slow inflammation and ulceration will be either speedily or eventually produced; and thus it is that pressure may induce both the ulcerative and progressive absorption; but the ulcerative is much less frequently induced than the progressive.

A morbidly-increased growth of the "boney bridge" and its processes has been mentioned as one cause of the angular projection of the spine, but it is not the only one; for the spinous process must protrude whenever the thickness of the bodies of one or two vertebræ is so much diminished by absorption, that one vertebra, especially in its long diameter, permanently inclines over its centre, or axis, to its anterior half. For, as in their natural situation, most of the spinous processes slope downwards, and overlap the one immediately below, like the plaits of a coat of mail, so must one protrude outwards beyond its proper or spinal line, whenever it is raised by depressing the anterior portion or opposite end of the oblong bone of which it forms the extreme part or end; and, in proportion to the degree of its elevation, must it raise the spinous processes above it, and throw the weight of the column on the anterior parts of the bodies of the vertebræ. Of course, it is here supposed to be understood that the depression of the anterior portion of the vertebra cannot be effected without its thickness being diminished by absorption. It appears to me that when one or two vertebræ are thus partially absorbed, the angular projection is produced; but, if more vertebræ are implicated in the absorption, then a curvature ensues.

It should have been previously observed, that two kinds of increased growth of bone occur in the vertebræ, as in other bones,—one depending upon a ricketty constitution, in which the bones are generally but unequally enlarged, and the other upon any diseased action which occasions partial enlargement; but the one depending upon a ricketty constitution is by far of the most common occurrence in children.

It has been cursorily remarked, that rickets is the fruitful source of lateral curvatures. This disease is, indeed, but rarely the cause of any other species: all the anatomical preparations we have seen of specimens of lateral curvature have appeared to proceed from this cause; and all the cases of lateral curvature in the living that have fallen under our management and observation, except one, have elsewhere displayed most unequivocal marks of the ricketty growth of bone. Lateral curvatures, at their commencement, are frequently single, and, if neglected in this state, generally become double; and are said to resemble the Roman letter S by Mr. Lloyd and many other authors, the italic *ſ* by Mr. Wilson; but, were we to choose a resemblance from alphabets, we should state that, in fact, it bears a much stronger likeness to the Greek ς (sigma), from the lower curve being always smaller than the upper. Lateral curvature is sometimes, though rarely, combined with excurvation; a very few instances of which we have seen. A case of this kind is mentioned by Mr. Wilson, and a specimen of one may be found in Mr. Brookes's museum.

In lateral curvatures from rickets, the cervical vertebræ generally preserve the erect attitude, and with some of the lumbar vertebræ maintain their spinal line. In Mr. Brookes's museum, there is a rare and valuable specimen of an irregular growth of bone producing lateral curvature of the upper cervical vertebræ in an adult, without evincing any appearances of caries or of rickets. The first, second, and third cervical vertebræ are united to each other and to the occipital bone, by anchylosis, and a boney septum, of considerable size, is thrown across the commencement of the spinal canal. The line of curvature on the right side measures one inch three lines, and on the left side two inches four lines; so that there is a difference of one inch and one line between the inner and outer lines of curve, and consequently of the thickness of the vertebræ.

[To be continued.]

